# Oxygen in the neonatal ICU: a complicated history and where are we now?

**DOI:** 10.3389/fped.2024.1371710

**Published:** 2024-05-01

**Authors:** Rachna R. Mamidi, Cindy T. McEvoy

**Affiliations:** Division of Neonatology, Oregon Health & Science University, Portland, OR, United States

**Keywords:** hyperoxia, hypoxia, oxygenation, neonate, preterm infant, retinopathy of prematurity (ROP), neonatal intensive care unit (NICU)

## Abstract

Despite major advances in neonatal care, oxygen remains the most commonly used medication in the neonatal intensive care unit (NICU). Supplemental oxygen can be life-saving for term and preterm neonates in the resuscitation period and beyond, however use of oxygen in the neonatal period must be judicious as there can be toxic effects. Newborns experience substantial hemodynamic changes at birth, rapid energy consumption, and decreased antioxidant capacity, which requires a delicate balance of sufficient oxygen while mitigating reactive oxygen species causing oxidative stress. In this review, we will discuss the physiology of neonates in relation to hypoxia and hyperoxic injury, the history of supplemental oxygen in the delivery room and beyond, supporting clinical research guiding trends for oxygen therapy in neonatal care, current practices, and future directions.

## 1 Introduction

The transition from fetus to newborn is a complex adaptation that results in a low oxygen tension fetal environment to rapidly increasing oxygen exposure postnatally. One study in the 1950s found the oxygen tension of amniotic fluid in preterm infants to be very low, mostly between 6 and 14 mm Hg (1). The low resistance placenta is the source of fetal gas exchange and nutrition; however at birth, with the removal of the placenta, major changes in the cardiovascular system occur including decrease in pulmonary vascular resistance and subsequent increase in oxygen tension (2, 3). Furthermore, neonates have high-energy needs as rapid growth and development demand high oxygen delivery and consumption (2, 4). The brain in particular is undergoing rapid post-natal expansion and requires efficiently functioning mitochondria with a steady supply of oxygen to meet energy demands. Mitochondria coupled with oxygen are also responsible for immune response, calcium buffering, and regulating reactive oxygen species (ROS) production, which can trigger programmed cell death (5–8). A delicate balance exists between adequate oxygen for growth and development yet avoiding excess oxygen that can lead to toxicity and cell death.

Hypoxia occurs when there is inadequate oxygen at the cellular level and often presents as cyanosis (blue discoloration of skin and mucous membranes) in the neonate. Insufficient oxygen disrupts cerebral oxidative metabolism and can lead to depletion of energy reserves in tissues. Furthermore, hypoxia triggers neurotoxic biochemical pathways including: changes in membrane potentials and ion distribution, production of nitric-oxide, accumulation of excitatory amino acids in extracellular space, inflammation, and necrosis (9–11). During the hypoxic state, cells shift to anaerobic metabolism leading to buildup of lactate as a byproduct causing metabolic acidosis that further harms cellular processes. Chronic hypoxia ultimately leads to apoptosis and cell death, particularly in the brain resulting in a decreased number of neurons (9, 12). Hypoxia of the fetus or newborn is one of the main causes of brain injury that can lead to long-term neurodevelopmental impairment, especially in preterm infants (13, 14).

Although preterm and term neonates require high oxygen demand they are particularly susceptible to oxidative stress and injury. Oxygen is a key component in energy metabolism in the mitochondrial respiratory chain by being reduced to water. This process generates ROS and reactive nitrogen species (RNS), some of which are free radicals and highly toxic. They damage the structure of nucleic acids, lipids, and proteins leading to oxidative injury (15–19). Other ROS, such as hydrogen peroxide, is not a free radical but important for physiologic cell signaling that regulates pulmonary circulation and blood flow within the ductus arteriosus (19). During normal fetal-to-neonatal transition the increased exposure to oxygen causes a mild physiologic oxidative stress that is crucial for postnatal adaptation. However, oxidative injury results when there is severe oxidative stress due to an imbalance between generated free radicals and inadequate neutralization by antioxidant systems, especially with a burst of free radical generation. Pulmonary capillary endothelial and alveolar epithelial cells are targets for ROS injury causing hypoplasia, lung edema, hemorrhage, and deposits of collagen, elastin, and hyaline membrane (20–24). The developing gastrointestinal tract is also affected by hyperoxia which causes thickened ileal mucosa, fewer Paneth, goblet cells and vili, decreased secretory components, increased inflammation and fibrosis, and cell apoptosis (25–31). All of these changes are detrimental to the gastrointestinal tract's vital function in mucosal immunity. Hyperoxia exposure is also associated with development of retinopathy of prematurity (ROP), which is an eye disease due to abnormal blood vessel growth that can lead to blindness. Oxidative stress has been linked to dysregulated signaling pathways that cause ROP (32, 33).

Preterm neonates are at a further disadvantage for combating oxidative stress due to decrease antioxidant capacity from insufficient accretion of important minerals and vitamins during gestation (34). Additionally, preterm infants are often born at a developmental stage with inadequate expression of antioxidant defense in the lung (35). Plasma vitamin C concentrations are initially higher in pre-term neonates than term neonates but follow a rapid and sharp decline over the first few days of life (36, 37). Biochemical vitamin D deficiency is seen in both term and preterm neonates but is more profound in preterm neonates (38–41). A systematic review and meta-analysis demonstrated preterm and term neonates have vitamin E concentrations below the recommended levels at birth (42). Preterm and term neonates are also known to have vitamin A deficiency with multiple clinical trials evaluating the benefit of supplementation by measuring outcomes such as mortality, oxygen use, and growth, but have shown mixed results (43). The largest randomized controlled trial evaluating vitamin A supplementation (NeoVitA) for preventing bronchopulmonary dysplasia (BPD) or death in extremely low birth weight neonates has yet to publish results (44). There are also clinical trials evaluating supplementation of antioxidant enzymes in preterm and term neonates. Superoxide dismutase (SOD) was a promising enzyme for supplementation however there is conflicting evidence regarding long-term follow up (45–48). A Cochrane meta-analysis demonstrated that there is insufficient data to conclude SOD is efficacious in preventing chronic lung disease of prematurity (46).

Oxygen toxicity is not limited to molecular injury but may also cause reprogramming of normal lung development. Alveolar simplification and aberrant, disorganized lung vasculature has been demonstrated in preterm baboons (49, 50) and in term rodents exposed to hyperoxia at birth (51, 52). Early exposure to hyperoxia inhibits normal cell proliferation and angiogenesis, leading to altered lung development. The degree of altered lung development may explain the variation in severity of respiratory outcomes in surviving preterm infants.

Pulse oximetry is the most common noninvasive method of measuring saturation of oxygen in the blood and guides the clinical application of supplemental oxygen based on specific thresholds. When air is breathed into the lungs, oxygen is transported into capillaries that in turn send oxygen rich blood to the heart, which subsequently gets pumped to the rest of the body. It is important to remember that the pulse oximeter detects oxygen saturation of the blood and not the lung itself as the lung likely requires increased oxygen to maintain normal oxygen tension in the blood and delivery to other organs.

## 2 Use of oxygen in the delivery room

### 2.1 Historical background

The discovery of oxygen is accredited to Joseph Priestly in 1774, however a Polish alchemist named Michal Sedziwój acknowledged its full significance as early as 1604 (53). Oxygen was used in neonatal resuscitation as early as 1780 with the first publication of oxygen use in newborn resuscitation occurring in 1928 (54–56). By the 20th century it became the standard of care for asphyxiated newborns due to an unfounded belief in perinatal brain damage resulting from birth asphyxia (54, 55). The potential toxicity of oxygen was first noted in the late 1940s by the first published description of retrolental fibroplasia (RLF), an eye disease affecting preterm neonates, now known as ROP (57). A husband and wife ophthalmologist team at Johns Hopkins University further confirmed the progressive nature of RLF in 1948 (58). It wasn't until the 1950s that oxygen was identified as the offending agent causing RLF, leading to blindness in children (59, 60). This discovery led to a paradigm shift in limiting oxygen in the delivery room that likely led to increased morbidity and mortality of preterm and sick neonates (61). With the development of oxygen saturation monitoring in the 1980s, there was renewed interest in targeted oxygen therapy. Multiple studies have described normal increases in oxygen saturation that occurs in healthy term neonates and thus current neonatal resuscitation guidelines supports increasing oxygen saturation targets in the first 10 min of life (62–66).

### 2.2 Term neonates

Asphyxiated term neonates were treated with 100% oxygen in the delivery room as the standard of care by the 1960s (67). The International Liaison Committee on Resuscitation (ILCOR) formed the first international guidelines for newborn resuscitation in 1992 in which use of 100% oxygen was recommended (68). Since those initial guidelines it has become clear that many practices at that time were not evidence based (69). By the 1990s, studies in human neonates demonstrated adequate resuscitation of asphyxiated term neonates with use of 21% FiO_2_ and harm in neonates exposed to 100% FiO_2_ (70, 71). Ramji et al. demonstrated in a randomized controlled trial (RCT) of 84 asphyxiated neonates that room air is as effective as 100% oxygen. Furthermore, neonates in the room air group had higher Apgar scores and less time to first cry with no neurologic sequela at 28 days follow up (70). Saugstad et al. lead an international, multicenter RCT including 703 asphyxiated neonates that demonstrated they could be adequately resuscitated with room air and recovered quicker than neonates resuscitated with 100% FiO_2_, assessed by Apgar scores and time to first breath and cry (71). At further follow up, conducted by a pediatrician using a standardized examination at 18–24 months, there was no significant difference in neurodevelopmental outcomes between the two groups (72). Studies in the 2000s further illustrated the harmful effects of resuscitation with 100% FiO_2_. Vento et al. demonstrated in a RCT of 40 asphyxiated term neonates that neonates resuscitated with 100% FiO_2_ compared to room air exhibited biochemical findings associated with prolonged oxidative stress that persisted even after 4 weeks of life (73). Vento et al. also published similar findings of 830 neonates treated over 6 years at their institution that further supported their conclusions (74). The most complete systematic review and meta-analysis published in 2008 demonstrated that asphyxiated neonates resuscitated with room air compared to 100% FiO_2_ had lower mortality with a trend toward decrease risk of hypoxic ischemic encephalopathy (HIE), a type of brain damage caused by lack of oxygen to the brain during or shortly after birth in which diagnosis is made by a combination of physical exam, blood work, and details of labor and delivery (75). Furthermore, long-term follow up did not show significant difference in neurodevelopmental outcome (76). These studies demonstrated a reduction in mortality with resuscitation using room air without evidence of harm. Term infants who are under normal hypoxic state during the fetal-to-neonatal transition and are then exposed to high concentration of oxygen (reoxygenation) during resuscitation likely generate oxygen free radicals that may explain this poorer outcome (77). Additionally, animal and human studies have demonstrated a decrease in cerebral blood flow with hyperoxia, increasing the risk for ischemic injury (78, 79).

Major changes occurred in ILCOR guidelines for newborn resuscitation from 2005 to 2010 including recommendation to start resuscitation with room air rather than 100% FiO_2_ (80). More recently, a systematic review and meta-analysis in 2019 further supported room air being superior to 100% FiO_2_ during the initial resuscitation of the term neonates by reducing neonatal mortality (risk ratio [RR] = 0.73; 95% confidence interval [CI]: 0.57–0.94) (81). This growth in evidence solidified the current neonatal resuscitation guidelines that strongly recommend starting with 21% FiO_2_ and caution against 100% FiO_2_ in neonates ≥35 weeks' gestation (82, 83).

### 2.3 Preterm neonates

At birth, fluid in the alveoli is reabsorbed and replaced with air. A combination of mechanical, vasoactive, and neural factors along with increased oxygenation result in an increase in pulmonary blood flow. This allows the lungs to participate in gas exchange and supply oxygenated blood to the heart. Preterm infants are at a high risk of inadequate pulmonary blood flow due to immature architecture of the lung and surfactant deficiency thus they likely require a higher oxygen concentration during resuscitation than term infants. However, evidence for the optimal level of oxygen used in the resuscitation of preterm neonates is much less conclusive than in term neonates. By the 1990s there was evidence that use of high levels of oxygen may be unnecessary and harmful to preterm neonates (79). However multiple studies demonstrate 21% FiO_2_ is not sufficient for the preterm population (84–86). Therefore the optimal oxygen concentration in the initial resuscitation of preterm neonates is somewhere between 21% and 100% which is a broad range and tremendous knowledge gap in the field. The 2010 ILCOR consensus guidelines recommended against use of 100% FiO_2_ but initiating some supplemental oxygen in the initial resuscitation of preterm neonates and titrating based on oxygen saturation (87). A systematic review and meta-analysis in 2018 demonstrated no difference in mortality with use of lower (<40%) vs. higher (≥40%) FiO_2_ in the resuscitation of preterm neonates. Only 1 of the 10 included studies reported increased mortality in the lower (21%) vs. higher (100%) oxygen group (88). A RCT of 52 preterm neonates (<30 weeks' gestational age) demonstrated the higher (100%) oxygen group led to improved breathing effort, improved oxygenation, and shorter duration of mask ventilation as compared to the lower (30%) oxygen group without increased oxidative stress markers and no difference in mortality (89). There are plans for a prospective meta-analysis of current ongoing RCTs of neonates <29 weeks' gestation randomized to high (60%) vs. low (30%) oxygen for initial resuscitation at birth. Results projected to be complete by 2025 and neurodevelopmental outcomes by 2027 (90). The largest clinical trial to date of oxygen concentration for the resuscitation of preterm neonates in the delivery room demonstrated higher incidence of bradycardia in the room air group vs. 100% FiO_2_ (91). Studies further demonstrated oxygen saturation <80% at 5 min after birth was associated with increased mortality (92–94). The current consensus for the initial resuscitation of preterm neonates is to target an oxygen saturation of >80% by the first 5 min of life and avoid bradycardia with goal heart rate >100 beats per min.

## 3 Use of oxygen in the neonatal ICU beyond the initial resuscitation

### 3.1 Historical background

Hyperoxia (state of excess supply of oxygen in tissues and organs) beyond the initial resuscitation is associated with multisystem morbidity and mortality including lung injury (BPD), ROP, brain injury (intraventricular hemorrhage and periventricular malacia), and intestinal injury. Yet, the optimal concentration of oxygen is still unknown, especially in preterm neonates. When hyperoxia was identified as the cause of RLF and blindness in children in the 1950s, the use of supplemental oxygen was intensely curtailed by the 1960s despite estimate of 16 additional deaths for every case of blindness prevented (59, 95, 96). In 1977, a large prospective observational study could not identify a relation between high partial pressure of arterial oxygen (PaO_2_ ≥ 100 and 150) and RLF however demonstrated a strong association of RLF with cumulative supplemental oxygen exposure (97). With the advent of transcutaneous PO_2_ electrodes, oxygen monitoring became more accessible and precise allowing for tighter control by the 1980s and 1990s (98–100). In 2007, the American Academy of Pediatrics recommended an oxygen saturation range of 85%–95% in neonates in the first two weeks of life (101).

### 3.2 Oxygen saturation targets for preterm neonates

There were only a few, small, randomized trials evaluating oxygen saturation targets in preterm neonates until the early 2000s. The Supplemental Therapeutic Oxygen for Prethreshold Retinopathy of Prematurity (STOP-ROP) trial randomized 649 preterm neonates with prethreshold ROP at 35 weeks' postmenstrual age to either 89%–94% or 96%–99% oxygen saturation (SpO_2_) by pulse oximetry. This study demonstrated no difference in incidence of ROP, however the higher threshold group had more adverse pulmonary events (pneumonia and/or exacerbations of chronic lung disease and need for oxygen, diuretics, and hospitalization at 3 months corrected age) (102). The Benefits of Oxygen Saturation Targeting (BOOST) trial randomized neonates born <30 weeks' gestation who needed supplemental oxygen at 32 weeks' postmenstrual age to either 91%–94% or 95%–98% SpO_2_. This study demonstrated no difference in ROP, growth, or development; however the higher threshold group required more home oxygen (103). However, both these studies tested oxygen saturation targets that would be considered very high by today's standard.

Following these studies, the Neonatal Oxygenation Prospective Meta-Analysis (NeOProM) collaborative was formed in 2003 to investigate optimal oxygen targets in extremely preterm neonates (born at <28 weeks' of gestational age). This was a prospective multicenter study that included five clinical studies with similar protocols to provide individual participant data at trial completion for inclusion in an independent patient meta-analysis. This study randomized 4,965 preterm neonates <28 weeks' gestational age to either 85%–89% or 91%–95% SpO_2_ within the first 24 h of birth (104). The studies found no difference in combined outcome of death and/or major neurodevelopmental impairment at 18–24 months however the lower oxygen saturation target group had a significantly increased mortality (105–109). The lower target threshold group had reduced risk of ROP requiring treatment however there was significant heterogeneity with only one trial within NeOProM that found significant reduction in ROP treatment. Furthermore, this did not translate to increased severe visual impairment at 18–24 months (105–110). The lower oxygen target group also had higher incidence of necrotizing enterocolitis (NEC), a disease of the intestinal tract in which tissue lining becomes inflamed, dies, and sloughs off. However, the study demonstrated no difference between the groups for BPD, intraventricular hemorrhage (IVH), periventricular leukomalacia (PVL), and neurodevelopmental outcomes (108, 110). However, a major limitation to consider is the significant overlap in the actual exposed oxygen saturation between the groups resulting in poor separation of the intervention and comparison groups (110).

## 4 Methods of oxygen measurement and titration

Improved measurement of blood oxygen levels and more precise titration of supplemental oxygen may decrease the time spent in hypoxia and hyperoxia, thus reducing oxygen toxicity. In this section we will discuss current methods of oxygen measurement and titration and ongoing investigations in this area.

### 4.1 Pulse oximetry

#### 4.1.1 Principles

Measurement of arterial oxygen saturation by pulse oximetry is based on the attenuation of light to the materials through which the light travels and photoplethysmography, an optical technique used to detect blood volume changes in the microvascular bed (111). Red and infrared lights are used based on the differential absorption of oxyhemoglobin and deoxyhemoglobin. During systole, the transmitted light intensity decreases as light is absorbed by the hemoglobin in the arteries. During diastole, transmitted light intensity increases due to decrease absorption. The ratios of the absorbance of the two wavelengths of light during systole and diastole are compared by the pulse oximeter and a built in algorithm converts the ratios to pulse oximetry oxygen saturation (112). This is measured on the neonate by transmitting the light through a distal extremity with a detector placed on the opposite side of the same extremity. The major advantage of pulse oximetery is the non-invasive ease of application and ability for continuous monitoring, however many limitations exist.

#### 4.1.2 Limitations

Due to the shape of the oxygen-hemoglobin dissociation curve, pulse oximetry has difficulty detecting hyperoxia >90% SpO_2_. Therefore small increase in SpO_2_ may indicate large increases in partial pressure of oxygen in arterial blood (PaO_2_) (113, 114). This is a physiologic limitation that has major implications for oxygen toxicity and stress in preterm neonates. Additionally, data used to calibrate pulse oximetry come from healthy Caucasian adults with adult hemoglobin ranges that are correlated to arterial blood samples. This poses a major limitation for calibration for different skin types, ethnicities, and ages. When calibrating for SpO_2_ < 80%, manufacturers rely on extrapolation and thus are not as accurate. Most manufacturers claim an accuracy of ±2% (115). Signal averaging time also places a limitation on pulse oximetry by causing response delays. Most conventional pulse oximeters have a default time of 8 s to display average values of SpO_2_ measured over this time period. Signal averaging time can be adjusted to a lower time however this may increase the frequency of alarms causing “alarm fatigue” to bedside providers. Averaging times can also be increased, however this may prevent detection of important hypoxemic episodes (116, 117). Motion artifacts are a common disturbance with pulse oximetry. This can lead to false alarms by movements of the probes or poor peripheral perfusion. However modern algorithms have decreased this issue. Despite these limitations, pulse oximetry remains the most widely used monitoring system in neonates. However, it is critical to recognize that pulse oximetry is a surrogate marker for tissue oxygenation and only measures peripheral arterial oxygen saturation.

#### 4.1.3 Automatic oxygen control

A major clinical variable affecting oxygen saturation targeting using pulse oximetry is the labor-intensive process of manually titrating FiO_2_ by bedside staff. Studies have shown despite multiple adjustments per hour, target ranges were achieved only 50% of the time (118). Based on this concern and need for tighter control of oxygen in neonates, automatic oxygen control (AOC) systems held high promise. AOC systems consist of an oxygenation monitoring device, (pulse oximeter), gas delivery system (ventilator or cannula), and an algorithm to determine timing and degree of FiO_2_ adjustments (119). Multiple studies, spanning the last 40 years, in term and very low birth weight preterm neonates have demonstrated AOC systems were more effective than manual titration in achieving oxygenation targets (120–133). There have been some studies that investigated long term effects and found no difference in neurodevelopmental outcome at 2 years of age between the automated oxygen control group and standard of care manual control groups (134).

Despite these findings there are many reasons AOC systems are not in widespread use. As the optimal target saturation for preterm neonates is still unknown a more consistent maintenance of target oxygen saturation may uncover adverse or beneficial effects. Furthermore, the reliability of pulse oximetry is key to a dependable AOC system. Close monitoring of this automated system is necessary to ensure accurate reading by pulse oximetry. Additionally, this automated system is not a replacement for provider response in investigating mechanism of oxygen saturation changes and appropriate intervention needed. Despite the known physiological benefits, there is insufficient data regarding improved clinical benefits to confidently introduce these systems into routine clinical care. There are currently ongoing trials to investigate effect of AOC on clinical outcomes including prolonged ventilation, death, severe ROP, BPD, NEC, and neurodevelopmental impairment (135, 136).

### 4.2 Near-infrared spectroscopy

Another technology that allows continuous non-invasive monitoring of oxygenation is near-infrared spectroscopy (NIRS). Unlike pulse oximetry, NIRS does not detect pulse waveform. It measures transparency of biological tissue of near-infrared spectrum of light affected by oxygen consumption and oxygen delivery by measuring ratio of oxygenation and deoxygenated hemoglobin (137). Originally developed to assess cerebral oxygenation perioperatively during cardiac surgery and neurosurgery, it has expanded to other clinical settings and ages including neonates. The most frequently measured sites in neonates include cerebral, renal, and splanchnic (intestinal) oxygenation.

#### 4.2.1 Cerebral NIRS

The brain is especially vulnerable to hypoxia during the fetal to neonatal transition and thus cerebral NIRS was considered a potential tool in resuscitation of newborns in the late 1970s (138). However given lack of established standards it has been difficult to include cerebral NIRS in the routine resuscitation of newborns. A systematic review in 2022 investigated whether early NIRS monitoring (<6 h of age) can predict neurodevelopmental outcome at 1–2 years in infants with HIE and demonstrated no significant difference in values of cerebral oxygenation. They further concluded very little data exists and further studies are required with standardized approach for adequate comparison (139). A recent multinational, randomized controlled trial of over 600 preterm neonates demonstrated no difference in survival or cerebral injury when cerebral NIRS was measured in combination with defined treatment guidelines in the immediate resuscitation after birth (140).

#### 4.2.2 Renal NIRS

Acute kidney injury (AKI) can result from low renal blood flow especially in the setting of a patent ductus arteriosus (PDA) in very low birth weight neonates. Renal NIRS has been used to help identify early markers of AKI. A prospective observational study in France demonstrated low renal NIRS values during the first 24 h of life is associated with development of AKI in preterm neonates (141). However conflicting evidence exists as well. High renal NIRS in neonates with HIE was demonstrated to be associated with AKI, whereas lower renal NIRS in neonates undergoing cardiac surgery was associated with AKI (142, 143).

#### 4.2.3 Splanchnic (intestinal) NIRS

There has been robust interest in the use of splanchnic or intestinal NIRS in newborns to detect early markers for development of necrotizing enterocolitis (NEC). A prospective cohort study of 100 preterm neonates demonstrated decreased intestinal NIRS values and increased variability in neonates who developed NEC (144). Another prospective observational study of 10 preterm neonates and 20 matched controls demonstrated intestinal oxygenation is impaired before the onset of clinical NEC (145).

#### 4.2.4 Limitations

NIRS is an indirect measure of oxygenation and therefore has several limitations of its application when there is movement, low arterial oxygen levels, darker skin pigmentation, or changes in weight and/or edema (146). Perhaps the biggest limitation is that there are no established standards and specific guidelines for use and interpretation of NIRS in neonates are lacking. Currently, there is not enough evidence to support the routine use of NIRS in neonates to justify additional monitoring on fragile neonates with limited body surface area.

## 5 Future directions

Currently, there are no available therapies to combat oxidative injury in neonates. Further research in antioxidant therapy including target specificity, bioavailability, and genetic variability may reveal novel approaches to mitigate oxygen toxicity. Stem cells hold therapeutic potential for hypoxia induced cellular injury and apoptosis by promoting neuronal cellular repair and regeneration. However more clinical studies are needed to determine the stem cell type, patient selection, route and time of administration to achieve standardized products and a refined protocol (147). Additionally the optimal level of positive end-expiratory pressure (PEEP) to minimize hyperoxic lung injury but provide adequate lung recruitment during invasive ventilation is unknown. Future research in ventilatory strategies that can be individualized through oxygenation guided lung recruitment methods may provide a personalized approach to mitigate effects of hypoxia and hyperoxia. Furthermore, noninvasive ventilation in preterm infants is also not standardized. Continuous positive airway pressure (CPAP) is the standard of care for preterm infants with respiratory distress syndrome, however the level and duration of CPAP is highly variable. Future research in this area may minimize lung injury by investigating the optimal CPAP strategies to reduce hypoxia and hyperoxia in preterm infants.

## 6 Conclusions

Term and preterm neonates face the unique dilemma of potentially needing life-saving oxygen supplementation while also at risk for oxygen toxicity. The rapidly changing physiology at birth, the extensive energy needs for growth and development, and decreased antioxidant capacity put them at this unique risk. Neonatology has had a complicated history with the application of oxygen from using it ubiquitously to fierce restriction due to retinopathy of prematurity, which left our sickest and most preterm neonates at risk of morbidity and mortality. With the advent of pulse oximetry there has been more refined approach to oxygen supplementation and titration however the exact thresholds still remain unknown. Furthermore, it is likely that optimal oxygenation saturation is not binary but varies throughout NICU hospitalization. There are ongoing investigations into automatic oxygen control and near-infrared spectroscopy to shed light on the optimal strategies for oxygen thresholds while avoiding oxygen toxicity and stress. Further research in antioxidant systems, stem cell therapy, optimal PEEP, and optimal CPAP level and duration may also reveal other strategies to improve oxygen therapy in preterm infants.
